# Mouse Nerve Growth Factor Injection and Progression Rate in Patients With Amyotrophic Lateral Sclerosis: An Observational Study

**DOI:** 10.3389/fneur.2022.829569

**Published:** 2022-02-17

**Authors:** Jia-Tong Li, Si-Qi Dong, Ting Qian, Wen-Bo Yang, Xiang-Jun Chen

**Affiliations:** ^1^Department of Neurology, Institute of Neurology, Huashan Hospital, Fudan University, Shanghai, China; ^2^National Center for Neurological Disorders, Shanghai, China; ^3^Human Phenome Institute, Fudan University, Shanghai, China

**Keywords:** amyotrophic lateral sclerosis, nerve growth factor, body mass index, ALSFRS-R, side effects

## Abstract

**Objectives:**

Amyotrophic lateral sclerosis (ALS) is a progressive, fatal disease with no curative treatment up to now. This study aims to analyze ALS progression of patients treated with mouse nerve growth factor (mNGF), as well as the effects, side effects, and adverse events of the therapy.

**Materials and Methods:**

A retrospective, observational study was performed including 94 patients with ALS from July 2020 to July 2021. Thirty-two of them were treated with at least one course of mNGF on a regular riluzole use, and the rest 62 were treated with riluzole only. The declining rates of body mass index (BMI) and ALS Functional Rating Scale-Revised (ALSFRS-R) scores were compared between the two groups to indicate ALS progression.

**Results:**

No significant differences in ALS progression indicated by the declining rates of BMI and ALSFRS-R score were observed between the two cohorts. ALS progression before and after the first treatment course of mNGF also showed no discernible difference. However, we noticed a moderate 62.7 and 25.1% reduction in the declining rate of BMI and ALSFRS-R motor subscore when comparing mNGF + riluzole treatment to riluzole only. The mNGF treatment was overall safe and well-tolerated, and a rare case of diarrhea was reported after mNGF injection.

**Conclusions:**

Our study revealed that mNGF treatment was overall safe and well-tolerated in patients of ALS. Application of mNGF combined with regular riluzole treatment had no significant clinical effects on delaying ALS progression. Prospective cohort studies and randomized clinical trials based on larger cohorts and longer follow-up times are needed to make a more convincing conclusion.

## Introduction

Amyotrophic lateral sclerosis (ALS) is a motor neuron neurodegenerative disease. It is characterized by progressive weakness, atrophy, and spasticity of muscles, which is a consequence of progressive degeneration of both upper and lower motor neurons (1). ALS exhibits a fatal, progressive disease course, with most patients dying 3–5 years after disease onset or within 15–20 months after diagnosis, mainly due to respiratory failure (1, 2). However, up to now, there has been no curative treatment for this devastating disease, except for two American Food and Drug Administration (FDA)-approved drugs, riluzole, and edaravone, which only show minimal effects (3–5). In recent years, many clinical trials have been devoted to finding effective therapies for ALS, such as drugs such as pramipexole (6), memantine (7), flecainide (8), and cellular therapies such as stem cell transplantation (9). Unfortunately, no significant progress in treating ALS has been achieved until now.

Neurotrophins (NTs) are a family of secreted proteins essential for regulating neuronal development, proliferation, differentiation, and maturation of both the central and peripheral nervous system (CNS and PNS), such as nerve growth factor (NGF), brain-derived neurotrophic factor (BDNF), glial cell-derived neurotrophic factor (GDNF), neurotrophin-3 (NT3), and neurotrophin-4 (NT4) (10). These NTs not only share a highly homologous sequence, but are also highly conserved across species, such as humans, mice, and rats (11). NGF is an endogenous NT composed of a 2:1:2 proportion of α, β, and γ peptide chains and was first discovered in the early 1950s (12, 13). NGF is produced by targeted tissues to provide protective and/or regenerative effects to neurons of the CNS and PNS *via* activating specific receptors at the axon terminals (14, 15). Mouse NGF (mNGF) is a biologically active protein extracted from the submaxillary gland of mice (16). In China, mNGF has been on the market for nearly 30 years and has officially been approved for treating peripheral nerve injury since January 2003 as the national level of class-A (13). Apart from providing neuroprotective and neuroregenerative effects in peripheral nerve injury (13), mNGF was also applied in treating CNS impairment such as Alzheimer's disease (17, 18), Parkinson's disease (19), severe traumatic brain injury (20), spinal cord injury (21) and hypoxic-ischemic brain damage (22, 23), ophthalmic diseases, such as glaucoma (24) and neurotrophic keratitis (25), and various other aspects, for instance promoting cardiac repair after myocardial infarction (26) and relieving early onset hearing loss (27). However, to our knowledge, no clinical report or study has examined the use of mNGF to treat patients with ALS who are characterized by the degeneration and impairment of motor neurons.

In the current study, we aimed to investigate the potential therapeutic effects of mNGF injection in treating ALS by retrospectively comparing patients treated with mNGF on the basis of regular riluzole use, and patients treated with riluzole only. Besides, we also monitored the side effects and adverse events of mNGF injection, hoping to assist clinicians to select a better therapeutic plan for ALS treatment.

## Materials and Methods

### Participants

Ninety-four patients diagnosed as definite or probable ALS according to revised El Escorial criteria (28) in our department from July 2020 to July 2021, were included in this study. Considering the different etiologies, clinical characteristics and prognoses, no familial patient with ALS was included. Ethical approval was given by the Ethical Committee of Huashan Hospital, Fudan University and medical records were obtained after informed consent from all the participants. Of the 94 participants, 62 were treated only with riluzole and were referred to as the “Riluzole Group.” The rest 32 patients accepted at least one course of mNGF injection on the basis of regular riluzole use and were defined as the “Riluzole + mNGF Group.” All participants were treated with riluzole (50 mg) two times a day for more than 2 weeks on a regular basis (29). mNGF (18 μg in 2 ml saline, Enjingfu, Sinobioway Biomedicine Co., Ltd, Xiamen, China) was injected intramuscularly for consecutive 28 days as one treatment course with a 6-month interval before the next course (30, 31).

### Study Design

The present study is a retrospective and observational study. Clinical parameters were retrieved from medical records of these participants as baseline information, such as age, gender, definite/probable ALS, disease duration, diagnosis delay, body mass index (BMI), onset domain, ALS Functional Rating Scale-Revised (ALSFRS-R) score, and pulmonary function. Disease duration was defined as the period between the onset of the first symptom to the baseline time. Diagnosis delay referred to the time interval between the onset of the first symptom and first-time precise diagnosis of definite/probable ALS. BMI was calculated as the weight in kilograms divided by the height in meters squared (32). ALSFRS-R is a validated scale to evaluate the functional impairment in the patients with ALS with a range from 0 to 48 points, with more impaired functions correlating with lower scores. The scale is divided into 4 subscores, assessing the bulbar, fine motor, gross motor, and respiratory function, respectively, with 12 points each (33). In this study, fine motor and gross motor subscores were added together to form a “motor” subscore for convenient analysis. Pulmonary function was assessed *via* forced vital capacity (FVC) and reported as a percentage of the predicted value (FVC %) (34).

All the BMI and ALSFRS-R score data were retrieved from baseline time to the last record time before July 2021 from the medical records of all participants, such as the data before and after the first use of mNGF. As other clinical parameters, such as FVC %, included a large amount of missing data, ALS progression was assessed by the declining rate of BMI and ALSFRS-R score in our study.

### Statistical Analysis

Shapiro–Wilk test was used to test the normality of continuous variables. Continuous variables were presented as means ± *SDs* for normal distribution and medians [interquartile ranges (IQRs)] for non-normal distribution. Numbers (%) were presented for categorical variables. Student's *t*-test and Wilcoxon Mann–Whitney test were performed to assess the difference between groups for normally distributed and non-normally distributed continuous variables, respectively. For categorical variables, Pearson's chi-squared test or Fisher's exact test was applied. Mixed effects models were used to estimate the change of BMI and ALSFRS-R score over time. The interaction effect between treatment and time, as well as the major effect of different treatments, were analyzed. A two-sided *p* < 0.05 was considered statistically significant. All the statistical analyses were performed using SPSS (Version 20, IBM, NY, USA).

## Results

### Baseline Characteristics

In this study, 94 patients who met definite or probable ALS diagnostic criteria were included. Thirty-two of them were treated with both riluzole and mNGF, and the rest 62 patients received only riluzole treatment (Table 1). No significant difference was observed when comparing the demographic information and clinical characteristics of the two groups. Some prognostic indicators of ALS, such as BMI, onset domain, ALSFRS-R score, and FVC % were also compared at baseline and no significant difference was observed.

**Table 1:** Demographic and clinical characteristics of patients at baseline.

	**Riluzole (*n* = 62)**	**Riluzole + mNGF (*n* = 32)**	**Riluzole + mNGF Group 1 (*n* = 7)**	**Riluzole + mNGF Group 2 (*n* = 11)**	***P*-value^†^**	***P-*value^‡^**	***P*-value^§^**
Age, years	58.9 ± 9.8	57.3 ± 9.1	57.4 ± 7.7	55.7 ± 8.5	0.430	0.702	0.317
Males	36 (58.1%)	16 (50.0%)	4 (57.1%)	6 (54.5%)	0.456	1.000	1.000
Definite ALS	27 (43.5%)	10 (31.2%)	2 (28.6%)	2 (18.2%)	0.247	0.721	0.211
Disease duration, months	19.5 (12.8, 29.3)	21.0 (15.3, 33.0)	30.0 (16.0, 55.0)	28.0 (18.0, 38.0)	0.176	0.056	0.054
Diagnosis delay, months	11.0 (6.0, 17.5)	12.0 (7.0, 23.0)	23.0 (8.0, 43.0)	12.5 (7.5, 27.0)	0.313	0.110	0.385
BMI	23.1 ± 2.8	24.3 ± 2.2	23.0 ± 2.2	24.2 ± 2.3	0.059	0.878	0.279
Bulbar onset	13 (21.3%)	4 (12.5%)	1 (14.3%)	2 (18.2%)	0.296	1.000	1.000
ALSFRS-R	40.0 (35.3, 43.8)	39.0 (36.0, 43.5)	40.0 (39.0, 43.0)	40.0 (38.0, 42.0)	0.979	0.766	0.962
FVC, % of predicted	80.6 ± 22.9	84.8 ± 19.2	80.7 ± 14.7	87.3 ± 17.6	0.447	0.993	0.376

*ALS, Amyotrophic Lateral Sclerosis; ALSFRS-R, ALS Functional Rating Scale-Revised; BMI, body mass index; FVC, forced vital capacity; mNGF, mouse nerve growth factor*.

*p-value^†^: Riluzole vs. Riluzole + mNGF*.

*p-value^‡^: Riluzole vs. Riluzole + mNGF Group 1*.

*p-value^§^: Riluzole vs. Riluzole + mNGF Group 2*.

### Comparison of ALS Progression Rate in Relation to Riluzole/Riluzole + mNGF Treatment

Considering the missing data of our patients with ALS cohorts, not all patients in the initial Riluzole + mNGF Group were included in further studies. Seven out of 32 patients with relatively complete BMI data records before and after the first use of mNGF were selected for BMI change analysis (referred to as “Riluzole + mNGF Group 1”). Similarly, “Riluzole + mNGF Group 2” contained 11 patients possessing detailed ALSFRS-R score data. The comparison of the demographic and clinical characteristics at baseline among Riluzole + mNGF Group 1, Riluzole + mNGF Group 2, and Riluzole Group showed no significant difference (Table 1).

The progression of ALS can be quantified by the rate of decline of clinical indicators. In this study, we used the rate of decline of BMI and ALSFRS-R score, which was calculated as the average decline per month, to indicate the progression of the disease (Table 2). Compared to patients with only riluzole treatment, Riluzole + mNGF Group 1 showed a 62.7% reduction in the rate of BMI decline, although the result was not significant [Riluzole Group: −0.102 (−1.906 to −0.013) vs. Riluzole + mNGF Group 1: −0.038 (−0.191–0.116); *p* = 0.579]. ALSFRS-R score also revealed an insignificant 5.2% reduction in declining rate in the group of patients treated with riluzole + mNGF [Riluzole Group: −1.117 (−1.952 to −0.283) vs. Riluzole + mNGF Group 2: 1.059 (−2.141–0.024); *p* = 0.457]. ALSFRS-R scale was then divided into three subunits, evaluating bulbar, motor, and respiratory function, respectively. No significant difference was observed in the rate of decline of the three ALSFRS-R subscores between Riluzole Group and Riluzole + mNGF Group 2, although bulbar subscore and motor subscore showed 4.3 and 25.1% reduction in declining rate, respectively (Table 2).

**Table 2:** Comparison of treatment effects between different groups.

**Outcome measure**	**Mean monthly rate of change**			***P*-value**
	**Riluzole (*n* = 62)**	**Riluzole + mNGF Group 1 (*n* = 7)**	**Riluzole + mNGF Group 2 (*n* = 11)**	
BMI	−0.102 (−1.906 to −0.013)	−0.038 (−0.191 to 0.116)	–	0.579
ALSFRS–R	−1.117 (−1.952 to −0.283)	–	−1.059 (−2.141 to 0.024)	0.457
ALSFRS-R bulbar subscore	−0.164 (−0.295 to −0.034)	–	−0.157 (−0.329 to 0.015)	0.912
ALSFRS-R motor subscore	−0.849 (−1.629 to −0.069)	–	−0.636 (−1.331 to 0.059)	0.326
ALSFRS-R respiratory subscore	−0.104 (−0.282 to 0.074)	–	−0.266 (−0.668 to 0.137)	0.722

*ALSFRS-R, ALS Functional Rating Scale-Revised; BMI, body mass index; mNGF, mouse nerve growth factor*.

**Figure 1:**
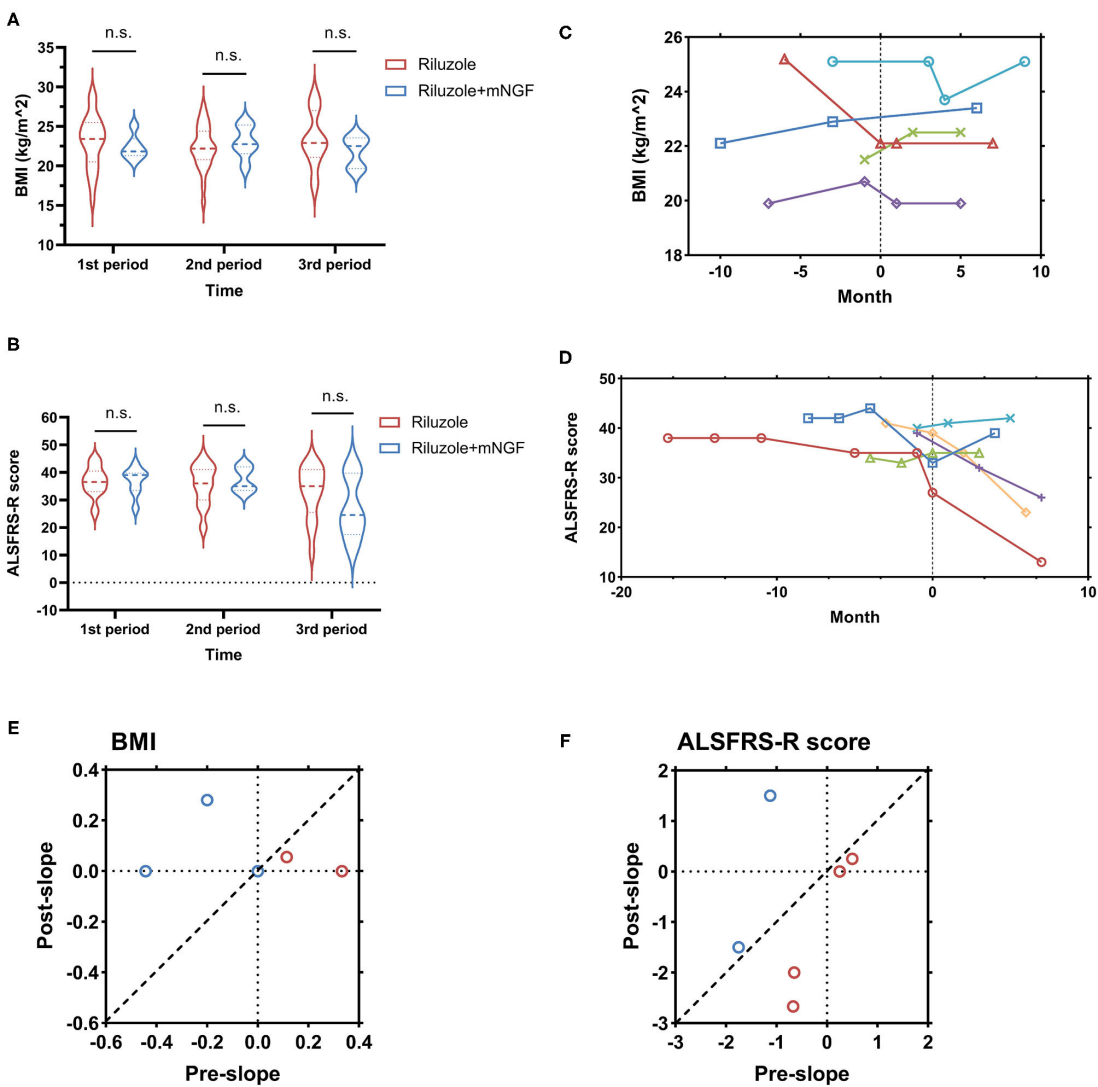
Comparison of the rate of decline of body mass index (BMI) and ALS Functional Rating Scale-Revised (ALSFRS-R) score between patients treated with mNGF + riluzole and patients treated with riluzole only. **(A,B)**, comparison of BMI **(A)** and ALSFRS-R **(B)** score within different time periods between the two treatment groups. “1st period,” “2nd period,” and “3rd period” refer to the 3 months before first mouse nerve growth factor (mNGF) injection, the 3 months after first mNGF injection and the 3–6 months after mNGF injection, respectively, in patients treated with mNGF + riluzole, and refer to the corresponding time periods in the riluzole only group; **(C,D)**, the change in BMI **(C)** and ALSFRS-R **(D)** score before and after the application of mNGF in several patients. Each curve of different color indicates an individual patient. “0” indicates the time point of the first mNGF injection; **(E,F)**, comparison of the declining rate of BMI **(E)** and ALSFRS-R **(F)** score before (indicated by the pre-slope axis) and after (indicated by the post-slope axis) the use of mNGF. Red circles indicate the increase of declining rate after mNGF treatment and blue circles indicate decreased or unchanged declining rate after mNGF treatment. ALSFRS-R, ALS Functional Rating Scale-Revised; BMI, body mass index; mNGF, mouse nerve growth factor; n.s., not significant.

In order to compare the time effect of different treatments, BMI and ALSFRS-R scores at different time periods were compared between the two treatment groups. For Riluzole + mNGF Group, BMI and ALSFRS-R data were retrieved and classified into three time periods: the 3 months before mNGF first use (1st period), the 3 months after mNGF first use (2nd period), and 3–6 months after mNGF first use (3rd period). As the first injection of mNGF was around October 2020 in most patients, BMI and ALSFRS-R data were collected for Riluzole Group in August 2020–October 2020 (1st period), November 2020–January 2021 (2nd group), and February 2021–April 2021 (3rd period) as a control. Neither BMI nor ALSFRS-R score revealed a significant difference at the three time periods between Riluzole Group and Riluzole + mNGF Group 1 or 2 (Figures 1A,B). Mixed effect models were conducted to assess the impact of different treatments on BMI and ALSFRS-R score across the three time periods. No significant interaction between treatment and time was observed for both BMI (*p* = 0.441) and ALSFRS-R score analysis (*p* = 0.403). The main effect of different treatments on either BMI or ALSFRS-R score was also insignificant (*p* = 0.399 and *p* = 0.542, respectively).

To further assess the individual change of BMI and ALSFRS-R score before and after the first use of mNGF, we compared the rate of decline of these indicators before (pre-slope) and after the use of mNGF (post-slope). Therefore, patients with at least three time points of clinical records before and after mNGF injection were selected from the Riluzole + mNGF Group for further analysis. The line charts illustrated changes with time of BMI and ALSFRS-R score for these individuals and obvious heterogeneity among patients was observed (Figures 1C,D). The “pre-slope” was calculated as the average slope of each curve before the time “0” point (first injection of mNGF). As one course of mNGF treatment was 28 days, the “post-slope” was defined as the average slope of each curve after time “1” point, which indicated the end of the first course of mNGF (Figures 1E,F). Interestingly, as shown in Figure 1C, the use of mNGF on the basis of riluzole treatment in some patients seemed to “stable” (the red and purple lines) or even “inverse” (the light blue line) the declining trend of BMI curve, although the result was not statistically significant (−0.039 ± 0.133 for pre-slope vs. 0.067 ± 0.054 for post-slope; *p* = 0.480). There was also no significant change in ALSFRS-R score declining rate before and after mNGF use according to the curves (−0.573 ± 0.343 for pre-slope vs. −0.736 ± 0.643 for post-slope; *p* = 0.828).

### Side Effects and Adverse Events of mNGF

Side effects and adverse events were collected for all 32 patients receiving mNGF treatment. Local pain at the injection site was the most frequent side effect of mNGF injection, which was reported in 20/32 (62.5%) patients. However, the pain could be gradually relieved after the treatment course. Four out of 32 patients (12.5%) reported intermittent headaches and dizziness, which also disappeared in a short period after mNGF use. A rare adverse event was reported in a 35-year-old male patient, who experienced diarrhea up to more than ten times per day after the first injection. The stool was normal in shape and the patient did not have abdominal pain or any other gastrointestinal symptom. The patient also reported a chronic diarrhea history of 4–5 times per day before the onset of ALS. A colonoscopy was applied; however, no significant intestinal abnormality was observed. The patient stopped mNGF injection and the frequency of diarrhea decreased accordingly. In spite of the clinician's suggestion to stop the mNGF treatment, they continued the injection several days later and their diarrhea symptom was relieved 4–5 times per day. No patients treated with the first course of mNGF reported any remarkable adverse events that led to the discontinuation of the injections. Not any significant adverse events were observed after the first course of mNGF treatment.

## Discussion

Despite the fatal nature of ALS, effective treatment for it still remains a great challenge. In this retrospective, observational study, mNGF intramuscular injection was applied to the patients with ALS who were also on a regular treatment of riluzole to analyze the potential effect of mNGF application. Our results found no significant difference in ALS progression between patients accepting both mNGF and riluzole, and those who were treated with riluzole only. The monitor on the side effects of mNGF injection also revealed that the treatment was relatively safe and well-tolerated by the patients with ALS.

In the present study, we retrospectively compared the patients with ALS who were treated with mNGF for at least one course on a regular riluzole treatment with those treated with riluzole only. Many previous studies have confirmed the effectiveness and safety of mNGF in treating various impairments of the CNS and PNS (35). Previous studies have shown that intramuscularly injected NGF can be transported retrogradely along the axons, which was characterized by rapid and unidirectional movements (15). Several hypotheses may explain the mechanisms involving the central and peripheral effects of exogenous NGF. First, NGF may arrest the ongoing degeneration of cholinergic projections to the cerebral cortices and promote the sprouting of new cholinergic axon terminals (36). Second, NGF provides neurotrophic effects by upregulating nicotinic receptors in the CNS, which will lead to a vasodilatory effect and improve global neuronal metabolism (37, 38). Third, exogenous NGF combines with high affinity NGF receptor TrkA and low affinity p75-NTR receptor, increasing the synthesis and excretion of endogenous NGF, which accelerates the mature of regenerative axons (39). Fourth, NGF can also elicit a cAMP-mediated decrease in RhoA which subsequently activates the regeneration-associated genes (RAGs), therefore, shifting the balance to an active regenerative state from the “regenerative brake” (40). Unfortunately, our study revealed no statistically significant difference in ALS progression, which was indicated by the rate of decline of BMI and ALSFRS-R score, between the two groups of patients. In addition, no discernible change in the declining rate was observed before and after the first course of mNGF treatment. However, other reasons that may contribute to the insignificance cannot be excluded: (1) we noticed a moderate 62.7% decrease in the rate of BMI decline when comparing mNGF+riluzole treatment to riluzole only. Besides, the motor subscore of the ALSFRS-R scale also showed a 25.1% reduction of declining rate in the mNGF + riluzole treatment group. It is possible that the relatively small number of patients in our study influences the power of statistical tests. (2) we only included the first course of mNGF treatment for analysis. However, as the repair of neural damage is a slow process, we cannot exclude the possibility that mNGF might exhibit effectiveness after several treatment cycles.

When introduced in 18 μg dose per day, mNGF injection was well-tolerated and safe without any report of an adverse event that leads to discontinuity of the treatment. Interestingly, a rare adverse event of diarrhea was reported in a 35-year-old male patient, who exhibited no discernible impairment of the intestinal on colonoscopy. A previous study showed that exogenous NGF injection to the rats significantly enhanced the myoelectric activities in the colon, which may result in the fast transmission of gastrointestinal contents, probably leading to diarrhea due to unabsorbed water (41). Accordingly, a study in humans also revealed decreased colonic transit time and increased stool frequency after exogenous neurotrophic factor supplement (42). Considering the fact that the patient's diarrhea was relieved when pausing the use of mNGF, it is quite possible that mNGF was indeed the main cause of aggravated diarrhea in this individual patient. Noticeably, constipation seems to be a relatively common clinical symptom of patients with ALS due to the poor mobility of the gastrointestinal tract, as one study reported a 46% constipation rate in patients with ALS higher than the normal population (43). Under this circumstance, it is considered to evaluate the potential therapeutic effect of mNGF in treating the patients with ALS accompanied with refractory constipation and this hypothesis needs further exploration.

The results of this study should be viewed in light of some limitations. First, due to the observational and retrospective nature of our study, the results may be blurred by various confounding factors and the causal relationship between mNGF treatment and ALS progression cannot be elucidated. Second, the number of patients involved in the present study was relatively small, and only the first course of mNGF treatment was included for analysis. Therefore, further prospective cohort studies and randomized clinical trials based on the larger cohorts and longer follow-up times are needed to provide more convincing results. Third, we were a lack in complete follow-up data of pulmonary function and electrophysiological examination, which were both important indicators for assessing ALS progression. Despite the limitations above, our study preliminarily suggested that mNGF had no significant beneficial effects on delaying ALS progression. However, a moderate reduction of the declining rate of both BMI and motor function was noticed in patients with the first course of mNGF, which may provide some insight into further clinical trials for ALS treatment.

## Conclusions

In this preliminary study, we investigated the potential effects of mNGF injection on patients with ALS. Application of mNGF combined with regular riluzole treatment had no significant clinical effects on delaying ALS progression, despite a moderate reduction in the declining rate of BMI and motor function. Therefore, prospective cohort studies and randomized clinical trials based on larger cohorts and longer follow-up times are needed to make a more convincing conclusion.

## Data Availability Statement

The original contributions presented in the study are included in the article/supplementary material, further inquiries can be directed to the corresponding author/s.

## Ethics Statement

The studies involving human participants were reviewed and approved by the Ethics Committee of Huashan Hospital, Fudan University. The patients/participants provided their written informed consent to participate in this study. Written informed consent was obtained from the individual(s) for the publication of any potentially identifiable images or data included in this article.

## Author Contributions

J-TL and X-JC contributed to the conception, design of the study, and wrote the manuscript. J-TL, S-QD, and TQ contributed to the analysis and interpretation of data. All authors collected clinical data, revised, and approved the manuscript.

## Funding

Funding for this study is provided by the 2020 Central Transfer Payment Medical Siege Institutions Capacity Building Project (National and Provincial Multi-scientific Cooperation Diagnosis and Treatment of Major Diseases Capacity Building Project) and the Shanghai Municipal Science and Technology Major Project (2018SHZDZX01) and ZJLab.

## Conflict of Interest

The authors declare that the research was conducted in the absence of any commercial or financial relationships that could be construed as a potential conflict of interest.

## Publisher's Note

All claims expressed in this article are solely those of the authors and do not necessarily represent those of their affiliated organizations, or those of the publisher, the editors and the reviewers. Any product that may be evaluated in this article, or claim that may be made by its manufacturer, is not guaranteed or endorsed by the publisher.
